# Roles and experiences of nurses in primary health care during the COVID-19 pandemic: a scoping review

**DOI:** 10.1186/s12912-024-02406-w

**Published:** 2024-10-11

**Authors:** Bada Kang, Eui Geum Oh, Sue Kim, Yeonsoo Jang, JiYeon Choi, Kennedy Diema Konlan, Hyeonkyeong Lee

**Affiliations:** 1 WHO Collaborating Centre for Research and Training for Nursing Development in Primary Health Care, College of Nursing, Yonsei University, 50 Yonsei-ro, Seodaemun-gu, Seoul, 03722 Republic of Korea; 2 Mo-Im Kim Nursing Research Institute, Yonsei University College of Nursing, Seoul, Republic of Korea; 3 Yonsei Evidence Based Nursing Centre of Korea: A JBI Affiliated Group, Seoul, Republic of Korea; 4 Department of Public Health Nursing, School of Nursing and Midwifery, University of Health and Allied Sciences, Ho, Volta Region Ghana

**Keywords:** COVID-19, Pandemic, Nurses, Roles, Experiences, Primary health care

## Abstract

**Background:**

Nurses form the frontline of the healthcare system’s response to both epidemics and pandemics, and this was especially the case during the novel coronavirus disease (COVID-19) pandemic. Although the influence of COVID-19 on nursing roles has attracted interest, there is no integrated knowledge of nurses’ roles and experiences in primary health care settings during the COVID-19 pandemic. Thus, this study identifies the roles and experiences of nurses in primary health care during the COVID-19 pandemic.

**Methods:**

A scoping review study design and the Joanna Briggs Institute methodology were used. The study searched five electronic databases (PubMed, CINAHL, EMBASE, Scopus, and PsychINFO) and included studies published in English from March 2020 to June 2023 that focused on the roles and experiences of nurses (*participants*) during COVID-19 (*concept*) in primary health care settings (*context*).

**Results:**

Fourteen articles were selected for review, involving a total of 1,487 nurses as study participants. The various roles undertaken by nurses in primary health care settings were categorized as comprehensive care providers, supporters and empowerers, coordinators and collaborators, information navigators, and change agents. Challenges and strategies are multilevel intrapersonal, interpersonal, organizational, community, and societal issues, but are not mutually exclusive.

**Conclusions:**

The pandemic-induced challenges revealed primary health care nurses’ vital and indispensable roles and resilience. They also fostered a heightened awareness of technological influence on the progression of primary health care in the current milieu. Policymakers and healthcare organizations need to integrate primary health care nurses’ expanding and emerging roles within the scope of practice, ensuring their effective implementation without excessive regulatory constraints. This study emphasizes the importance of developing multilevel interventions to address the support needs of primary health care nurses through a system-based approach. Building a strong infrastructure to support nurses’ self-care, offering continuing professional development opportunities, and securing official government recognition will be essential for enhancing the resilience of primary healthcare nurses in preparation for future, potentially devastating pandemics.

**Supplementary Information:**

The online version contains supplementary material available at 10.1186/s12912-024-02406-w.

## Background

The coronavirus disease (COVID-19) pandemic has profoundly impacted healthcare systems at the global, national, and local levels [1, 2]. While countries struggled to respond to the rapid surge in COVID-19 cases during the early stages of the pandemic, primary health care (PHC) was called on to take immediate action to contain the virus as the foundation for an efficient global response to the pandemic [3]. As such, attention has been paid to PHC globally as the healthcare system’s first line of defense and gatekeeper. The significance of PHC based on health equity and fairness has been further underscored during the COVID-19 crisis. Moreover, in the wake of the pandemic, the nursing profession faces the challenge of continuing its efforts to strengthen the foundation of PHC.

Furthermore, today’s healthcare systems confront other major challenges imposed by an aging population and increased non-communicable diseases [4, 5]. While a significant reduction in premature mortality due to communicable diseases has shifted the global disease burden to non-communicable diseases [6], communicable, maternal, neonatal, and nutritional diseases still account for more than 60% of the diseases in low-income countries [7, 8]. As such, the health and social care systems of low- and middle-income countries have faced challenges in mitigating the double burden of infectious and non-communicable diseases [9]. Moreover, international mobility has facilitated the global transmission of emerging and re-emerging infectious diseases (e.g., Severe Acute Respiratory Syndrome and Middle East Respiratory Syndrome) with global health significance [10], while the recent COVID-19 pandemic has added another layer of complexity [11].

PHC is widely regarded as the most effective and efficient approach to responding to complex emerging public health problems [12]. As primary care is a “first-contact, accessible, continuous, comprehensive and coordinated patient-focused care” in a health system, the scope of PHC includes all areas where primary care is provided, including the community’s first level of contact with the health system [12]. The 2018 Astana Declaration emphasized strengthening PHC to fulfill the health-related Sustainable Development Goals [13]. As the global burden of aging demographics and non-communicable diseases increases, PHC is expected to serve as the central platform providing a wide range of prevention and coordination of life-long chronic conditions, including promotive, preventive, curative, rehabilitative, and palliative services for all people throughout their lives [13, 14]. The COVID-19 pandemic has also stressed the public health functions of PHC, particularly in response to pandemics and epidemics [14, 15].

Nurses, the largest group of healthcare professionals delivering frontline care [16], have been essential during the COVID-19 pandemic [17]. Nursing roles and the scope of practice in PHC generally vary and are shaped primarily by service funding and the healthcare system in a particular country or geographic region [18]. These roles are described as independent, dependent (e.g., requiring a physician order), or interdependent, that is, collaborative delivery with other practitioners [19]. Despite the heterogeneity of roles and contexts of nursing at the international level [20], PHC nurses have been responsible for responding to the COVID-19 emergency while maintaining essential primary care services to meet the ongoing health needs of the community and overcoming a unique set of barriers imposed by the social distancing and quarantine measures implemented worldwide [21]. Therefore, the influence of COVID-19 has aroused interest in evaluating how it has influenced nurses’ roles in PHC settings.

Evidence of the impact of the COVID-19 pandemic on PHC and the workforce has also aroused interest in evaluating how the pandemic has influenced nurses’ roles and experiences in PHC settings. A previous integrative review identified the barriers and facilitators to implementing nurses’ roles in PHC settings; however, this review was not confined to the COVID-19 pandemic [20]. A brief literature review focused on nurses’ roles in providing care for patients with COVID-19. However, it did not distinguish between PHC and other healthcare settings, such as acute care [22]. To date, international literature on nurses’ roles and experiences during the COVID-19 outbreak lacks integrated research. As cultivating an adequately trained workforce is a fundamental prerequisite for advancing PHC [23], understanding the functions of nurses and their extent during the COVID-19 pandemic can provide evidence for the PHC system, given future pandemic crises.

## The review

### Aim

This study aims to identify the nurses’ roles and describe their experiences with PHC during the COVID-19 pandemic. The research questions are as follows:

1. What roles did nurses play in PHC settings during the COVID-19 pandemic?
2. What challenges and barriers did nurses experience in fulfilling their roles in PHC during the COVID-19 pandemic?
3. What strategies did the nurses in PHC settings employ to overcome challenges in fulfilling their roles during the COVID-19 pandemic?

### Methods

#### Design

This scoping review followed the Joanna Briggs Institute (JBI) methodology to explore the nature and breadth of emergent heterogeneous research by mapping and summarizing the literature and addressing the knowledge gap [24, 25]. The review also followed the Preferred Reporting Items for Systematic Reviews and Meta-Analysis Extension for Scoping Reviews (PRISMA-ScR) [26].

#### Search methods

Published articles were searched using the following five electronic databases: PubMed (MEDLINE), CINAHL, EMBASE, PsycINFO, and SCOPUS. The core elements of the inclusion criteria in the JBI Scoping Review methodology were adopted to design three categories of search syntax: nurses (*participants*), their roles and experiences during the COVID-19 pandemic (*concept*), and PHC (*context*). Given that roles and experiences are broad concepts, we did not restrict them to specific keywords.

The search strategy was developed with the assistance of a medical librarian using a three-step strategy to ensure a comprehensive search of the peer-reviewed literature [24]. In the first stage, we searched PubMed (MEDLINE) and CINAHL to identify keywords and subject headings and refine the search strategy. In the second stage, the search strategy using the identified keywords and index terms was translated into other databases. Finally, an additional manual search of the bibliographies of the included articles was performed to screen for additional articles not captured during the database search. The search was limited to articles published from March 2020, when the World Health Organization declared the COVID-19 pandemic [27], to June 30, 2023. Publication status was not restricted when retrieving relevant articles. There are no rigorous systematic search methods for gray literature, particularly for reports published by government and non-government organizations [28, 29], and it was not feasible to include non-English language sources. Consequently, the gray literature retrieved was likely an unrepresentative subset, which could have introduced bias [28]. To avoid this, only peer-reviewed studies were included in this review. The detailed search strategy for each database is provided in Appendix 1 (see Supplementary Table 1).

#### Eligibility criteria

The pre-specified inclusion criteria were also based on the PCC framework as follows. For the **participant** and **context**, all types of nurses (e.g., registered nurses, licensed practical nurses, advanced practice nurses, clinical nurse specialists, and midwives) who work in PHC settings were considered. Regarding the **concept**, we included all studies that reported roles, experiences, or responsibilities during the COVID-19 pandemic. In this study, *roles* were defined as the responsibilities, activities, and tasks carried out by nurses during their involvement with virtual care interventions, programs, or initiatives in PHC [19, 30].

Studies were excluded if they: (1) involved mixed participants and did not report results separately according to each population; (2) reported only patients’ experiences of service acquisition and utilization during the COVID-19 pandemic; (3) were published in languages other than English; (4) had insufficient information (e.g., study protocols, ongoing studies); or (5) were non-empirical studies (e.g., review papers, opinions, editorials, letters, perspectives, short communications, commentaries, and social and professional statements).

#### Screening and study selection

The literature search results were exported to EndNote 20 (Clarivate Analytics, Philadelphia, PA, USA) to manage and remove duplicates. The three-stage screening process consisted of title and abstract scanning, followed by a full-text review. All aspects of screening, including title, abstracts, and full-text reviews, were performed by two authors (*anonymized for review*) using pre-specified inclusion criteria. All discrepancies between the two reviewers were resolved by discussion with a third reviewer (*anonymized for review*).

#### Critical appraisal

Although critical appraisal is not mandatory for a scoping review, we performed a critical appraisal to identify gaps in existing literature regarding methodological rigor [31]. Mixed Methods Appraisal Tool (MMAT) version 2018 was used to assess the quantitative descriptive and qualitative studies [32]. The included studies were independently assessed by two authors (*anonymized for review*), and disagreements were resolved through discussion with a third reviewer (last author) when necessary. Because the purpose of this scoping review was to consolidate all available studies on the roles and experiences of PHC nurses during the COVID-19 pandemic, no articles were excluded based on quality.

#### Data extraction and evidence synthesis

Following the matrix method [33], one reviewer independently extracted the data, and a second reviewer verified the accuracy of the extraction. The extracted data included general study identifiers (i.e., author, year of publication, and country of publication), specifics of the study methods (i.e., design, sample, setting, data collection, and data analysis), core concepts relevant to this review, and outcomes of significance to the research questions. Any disagreements between the two reviewers were resolved through discussions among all authors.

The extracted data were then summarized in tabular format to map the core concepts of this review (roles, challenges, barriers, and strategies). Through the synthesis process, we found that the socioecological model for human development was helpful as an analytical framework to identify the domains of nurses’ experiences during the pandemic, including challenges and barriers to fulfilling their roles and strategies adopted to overcome challenges. Socioecological models indicate that human behaviors are affected by multiple factors, including intrapersonal, interpersonal, organizational, and societal factors, and are integrated into the dynamic interplay of these factors [34, 35]. Summary narratives were written to synthesize the selected studies across tables and identify the advances and gaps in the literature.

## Results

### Search outcomes

**Fig. 1:**
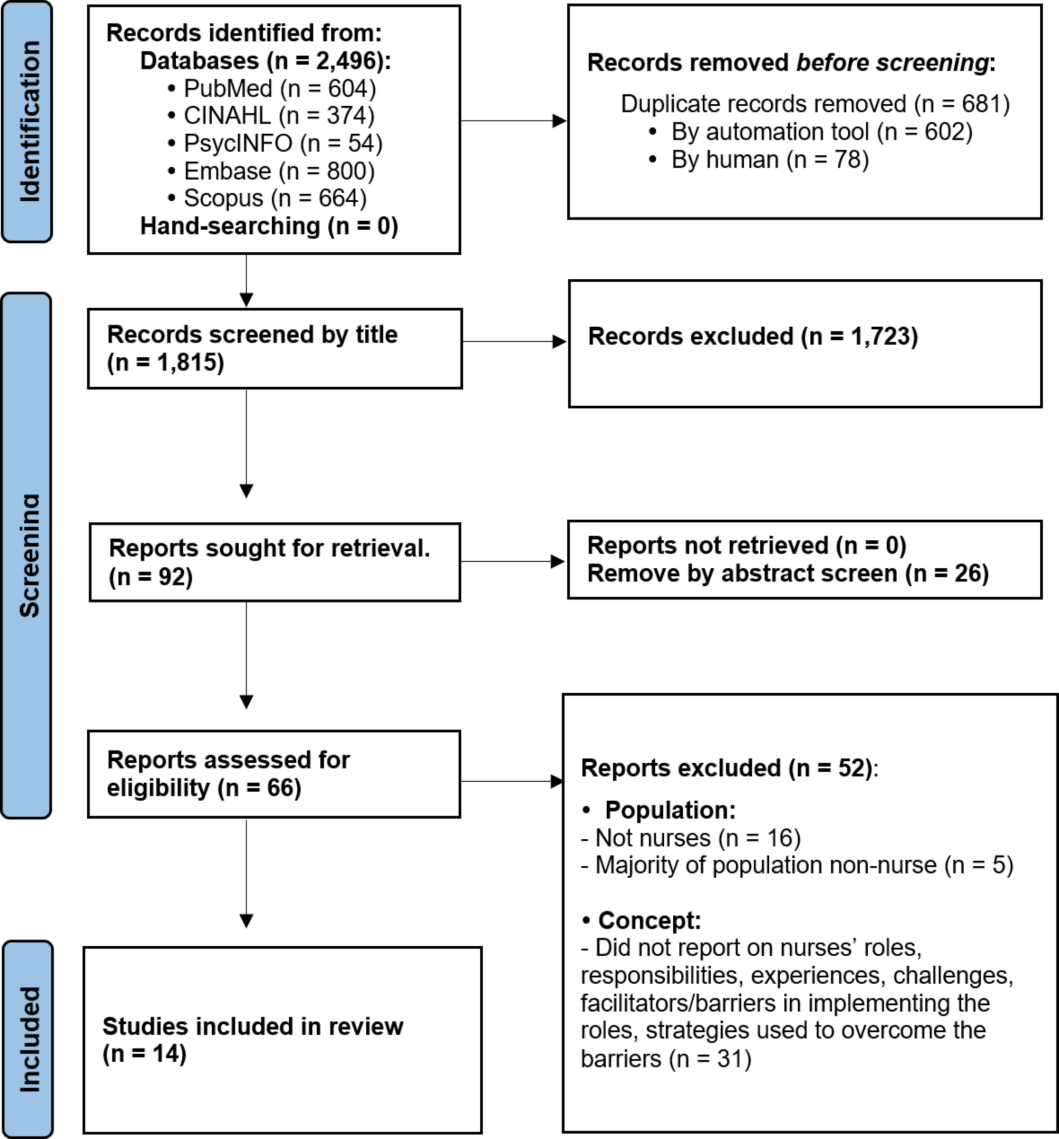
PRISMA Flowchart illustrating the study selection process

The PRISMA flowchart in Fig. 1 illustrates the screening process. The search yielded 2,496 articles. After duplicates were removed, 1,815 articles remained for review at the title and abstract levels. Of these, 66 were considered for full-text assessment. Finally, 14 studies met the inclusion criteria and were included in this review. Snowballing did not yield any relevant articles.

### Study characteristics

Table 1 presents the characteristics of the 14 included articles. Of these 14 articles, two sets (two articles in each) used the same dataset to address different aims [18, 36–38]. With publication years ranging from 2020 to 2023, 11 studies (78.6%) were published between 2021 and 2022. These studies were conducted in Asia (*n* = 4), Australia (*n* = 4), Africa (*n* = 3), and Europe (*n* = 3). All studies were cross-sectional and employed a qualitative design (*n* = 9) or a quantitative descriptive design utilizing a survey method (*n* = 5). Among the various PHC settings, most were community-based, including community health centers (*n* = 12), with two remaining studies conducted in outpatient departments that mainly delivered primary care in hospitals [39] and schools [40]. The participants in the included studies were registered nurses, enrolled nurses, nurse practitioners, midwives, school nurses, nursing assistants/associates, and community health volunteers.

**Table 1:** Main characteristics of the included studies

Author (Year)	Country	Purpose	Sample	Setting	Design/Data Collection
Adelekan et al. (2021)	Nigeria	To investigate how the COVID-19 pandemic and related lockdowns affected the provision of essential reproductive, maternal, child, and adolescent health services and the challenges in service delivery in PHC facilities	Head nurses and midwives (*n* = 307)	307 primary health centers in 30 local government areas in 10 states	Quantitative descriptive, a semi-structured interviewer-administered survey
Akbar et al. (2022)	Indonesia	To explore the community health nurses’ roles in the COVID-19 management	Community health nurses (*n* = 9) and community health volunteers known as health cadres (*n* = 2)	A city health office and 3 community health centers	Qualitative exploratory descriptive study, in-depth phone interviews
Crowley et al. (2021)	South Africa	To examine the preparedness of primary care nurses for COVID-19	Professional nurses enrolled for a Postgraduate Diploma in Primary Care Nursing and alumni working in primary care settings (*n* = 83, including 40 clinical nurse practitioners and 40 professional nurses)	32 public health clinics, 26 public community health centers, 5 public mobile clinics, and 16 other types of primary care settings	Quantitative descriptive, online survey (closed and open-ended questions)
Crowley et al. (2021)	South Africa	To examine the reorganization of primary care services during COVID-19 from the perspectives of primary care nurses	Professional nurses enrolled for a Postgraduate Diploma in Primary Care Nursing and alumni working in primary care settings (*n* = 83)	32 public health clinics, 26 public community health centers, 5 public mobile clinics, and 16 other types of primary care settings	Quantitative descriptive, online survey (closed and open-ended questions)
Halcomb et al. (2022)	Australia	To validate the safe and effective staffing tool and report on the perceptions of primary healthcare nurses on the impact of COVID-19 on the quality of care delivery	Nurses in primary health care settings (*n* = 359; 320 registered nurses, 30 enrolled nurses, and 6 nurse practitioners)	167 general practice, 97 community-based services, and 95 other types of primary health care settings	Quantitative descriptive, national online survey
Halcomb et al. (2020)	Australia	To investigate the experiences of nurses working in primary healthcare during the COVID-19 pandemic	Nurses in primary health care settings (*n* = 637; 555 registered nurses, 56 enrolled nurses, 22 nurse practitioners, 4 other)	351 general practice, 106 community-based services, and 180 other types of primary health care settings	Quantitative descriptive, online survey
Halcomb et al. (2020)	Australia	To identify primary healthcare nurses’ immediate support needs to enable them to provide quality care during the COVID-19 pandemic	Nurses in primary health care settings (*n* = 637 including registered nurses, enrolled nurses, and nurse practitioners)	351 general practice, 106 community-based services, and 180 other primary health care settings	Qualitative descriptive, in-depth qualitative open-ended responses to online survey questions
James et al. (2021)	Australia	To explore the experiences of primary healthcare nurses in the use of telehealth during COVID-19	Primary health care nurses (*n* = 25; 12 community-based nurses and 13 general practice nurses)	Diverse community-based and general practice settings	Qualitative descriptive study, semi-structured telephone interviews
Lee et al. (2021)	Hong Kong	To explore the experiences of school nurses during the COVID-19 pandemic in Hong Kon	School nurses (*n* = 9)	8 international schools, 10 special needs schools, and 1 private school	Qualitative descriptive study, semi-structured interviews
Martins et al. (2022)	Spain	To narrate the nurses’ experience of facing a long economic and political crisis and the process of the COVID-19 pandemic in primary care settings	Nurses (*n* = 10) and nursing assistants (*n* = 2)	A primary care unit in a healthcare center	A descriptive qualitative study with ethnographic analysis, in-depth interviews, participant observation, and field diary records
Mizumoto et al. (2022)	Japan	To explore how nurses working in primary care were psychologically and socially affected by the COVID-19 and how they overcame the difficulties and cope with the COVID-19	Nurses working in primary care (*n* = 7)	An outpatient department for family medicine of a small hospital, which mainly delivers primary care	Qualitative study, participants’ notes, recorded discussions, and participant’s written impressions before, during, and after a workshop
Nilsen et al. (2022)	Sweden	To explore lessons from the COVID-19 pandemic experienced by registered nurses and assistant nurses in primary health care	Registered nurses (*n* = 11) and assistant nurses (*n* = 10)	Primary healthcare centers (*n* = NR)	Qualitative study, semi-structured interviews via Zoom
Russsels et al. (2022)	England	To explore primary care nurses’ and healthcare assistants’ experiences and perceptions of general practice and the changes made to it during the COVID-19 pandemic	General practice nurses (*n* = 12), advanced nurse practitioners (*n* = 4), healthcare assistants (*n* = 7), and nursing associate (*n* = 1)	18 urban, suburban, and rural primary care settings	Exploratory qualitative study, semi-structured interviews via telephone or video call
Yodsuban et al. (2023)	Thailand	To describe the role and activities of community health nurses focusing on the care of older adults during the COVID-19 pandemic	46 key informants categorized into (1) public sector officers (*n* = 4 community health nurses, 1 = director of health promotion, 1 = public health staff), (2) public health staff of Local Administration Organization (*n* = 1), (3) Community leaders (*n* = 2 heads of the villages), (4) Civil groups (*n* = 16 health volunteers), (5) older adults (*n* = 22)	One specific sub-district in Northeastern Thailand was selected as a model area for outstanding community management practices of COVID-19	Qualitative descriptive study, in-depth interviews, field observations, secondary data, and focus group discussion using semi-structured interviews

NR = not reported

The included studies had three main purposes: (1) to examine the perception of PHC nurses regarding the impact of the COVID-19 pandemic on nurses or the nursing care process; (2) to describe the roles of PHC nurses during the COVID-19 pandemic; and (3) to explore the experiences of nurses working in PHC during the COVID-19 pandemic, including challenges and strategies.

### Critical appraisal of included studies

The critical appraisal of the included studies is presented in Appendix B (Supplementary Tables 1 and 2). Of the five quantitative descriptive studies, two [36, 37] recruited participants from a single university, limiting generalizability, while one study [41] was limited by selection and recall biases, given that a head nurse or midwife was interviewed as the only key informant per facility. Most quantitative descriptive studies were susceptible to nonresponse bias due to either low response rates [36, 37] or a lack of analysis on data missing at random [18, 38]. While the data collection method of most qualitative studies was appropriate, for one study [39], data collection was performed during participants’ duty hours at their workplaces, posing risks of biased responses. In terms of whether the findings were adequately derived from the data, in four studies [39, 42–44] the description of analysis methods or qualitative rigor was either not stated or clear. One study [43] provided quotes only in Spanish which can be a barrier to assessing whether the interpretation of results was sufficiently substantiated by data.

### Nurses’ role in PHC during the COVID-19 pandemic

Three qualitative studies involving 22 nurses explicitly focused on nurses’ role in PHC settings during the COVID-19 pandemic [40–45]. Additionally, two studies involving 108 nurses (*n* = 83 in the quantitative descriptive study and 25 in the qualitative study) indirectly highlighted the varied roles of nurses in PHC settings [37, 44]. Based on data from five studies involving 130 nurses that described nurses’ roles [37, 40, 42, 44, 45], the roles were divided into five main categories: (1) comprehensive care providers, (2) supporters and empowerers, (3) coordinators and collaborators, (4) change agents, and (5) information navigators.

#### Comprehensive care providers

Two studies showed that nurses assumed an expanded role as comprehensive care providers for COVID-19 patients alongside their existing responsibilities [45, 46]. Nurses also implemented a COVID-19 vaccination program and performed surveillance activities, including initial COVID-19 screening for health services and COVID-19 contact tracing in the community [45, 46]. They provided directions to patients and family members to self-isolate, either within their homes or in designated community quarantine facilities, and assessed COVID-19 symptoms and severity to ensure that the patients received suitable medical care [45, 46]. Addressing the needs of vulnerable groups, including patients with chronic conditions, remains a significant focus area for PHC nurses [45, 46]. Additionally, nurses conducted targeted home visits for patients experiencing deterioration in their condition and requiring specialized nursing care [45].

#### Supporters and empowerers

Nurses in PHC settings provided support and empowerment, contributing to both the local community and its organization. Nurses promoted the adoption of preventive measures by serving as role models and educating patients, fellow staff members, and the community [40, 45]. Nurses also offered counseling and support to address the psychological needs of fellow staff in their organizations [40]. Additionally, nurses empowered health volunteers to strengthen their capacity to manage COVID-19, including educating the community, tracing COVID-19 cases, and combating COVID-19-related social stigma in the community [45]. As patient advocates, they also supported patients to reintegrate from quarantine to mitigate social stigma in the community [46].

#### Coordinators and collaborators

Nurses coordinated and collaborated with pertinent organizations and network partners, both governmental and private, within and beyond the community, to efficiently manage COVID-19 prevention and control efforts, establish quarantine facilities, and advocate care continuity of COVID-19 patients by arranging transportation, referring patients for hospitalization, and streamlining the allocation of human resources [45, 46]. For example, nurses partnered with non-governmental organizations to implement training programs to enhance the capacity of community nurses and facilitate health education and surveillance training programs for residents. Additionally, nurses collaborated with the local COVID-19 task force to manage the pandemic by monitoring compliance with self-isolation [45].

#### Change agents

Five studies noted that nurses acted as change agents and implemented various initiatives to optimize services during the pandemic [37, 40, 42–44]. They acknowledged contextual challenges, such as limited space for social distancing, limited time and human resources, and staff resistance to wearing masks, and formulated appropriate responses, such as the reduction of lower-priority outreach programs [37, 40]. They also restructured the service-delivery model using telehealth and social media [44, 45] and established community-based COVID-19 control committees [45].

#### Information navigators

Some studies revealed that PHC nurses were crucial information navigators during rapidly evolving circumstances [40, 45]. Nurses proactively sought pertinent information and stayed up-to-date during the COVID-19 pandemic [40]. They also gathered and reported COVID-19 and other health-related data and contributed to formulating organizational protocols and guidelines [40, 45].

### Challenges and barriers to the implementation of PHC nurses’ roles

**Fig. 2:**
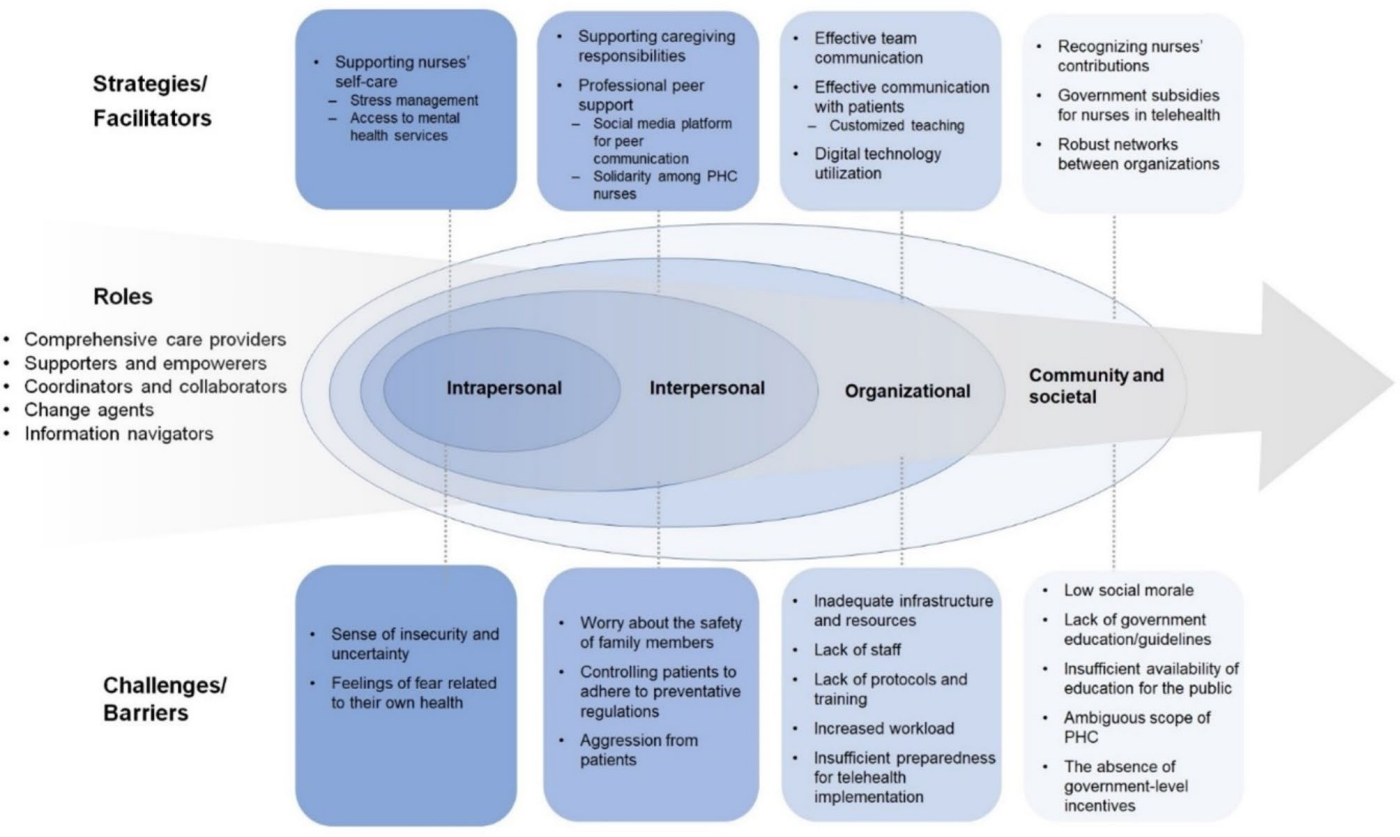
Strategies/facilitators and challenges/barriers, organized by socioecological framework

Our review identified the challenges and barriers to implementing various roles for PHC nurses, which can be categorized into four levels according to the socioecological framework (Fig. 2). While not mutually exclusive, these levels include the individual, interpersonal, organizational, community, and societal levels.

#### Individual level

Psychological distress related to unknown situations was identified as a challenge experienced by nurses in PHC settings in the literature. Nurses articulated feelings of fear of the unknown, a sense of helplessness, and experiencing anxiety and stress during the initial stages of the pandemic [39, 40]. An ethnographic study in Spain revealed insecurity and uncertainty regarding what to do and how to deal with COVID-19 in the initial stages of the pandemic [45]. In an online survey that examined the preparedness of nurses in South Africa for COVID-19, more than half of the participants reported a lack of confidence in the prevention and control of COVID-19 [36]. Additionally, data from four studies showed that nurses experienced feelings of fear related to their health, including the risk of exposure to and infection with COVID-19 and concerns about their underlying health conditions [18, 36, 41]. Job insecurity and decreased working hours further compounded the sense of insecurity [18, 38].

The adverse sentiments stemming from the COVID-19 pandemic were not consistent over time; instead, the initial sense of insecurity evolved into a state of resilience. Three studies noted a gradual transition from insecurity and uncertainty to preparedness and adaptability [39, 40, 47]. For example, in a qualitative study of nurses working in an outpatient department that mainly delivered primary care to the community in Japan, as nurses gained a sense of control over their tasks and responsibilities, they gradually cultivated a sense of professionalism while fulfilling their duty of caring for vulnerable patients [39].

#### Interpersonal level

At the interpersonal level, the main challenge faced by PHC nurses was addressing concerns about the health and safety of their family members because of the increased risk of exposure resulting from their work-related activities [18, 36]. Aside from their nursing responsibilities, their roles as family members were adversely affected by the circumstances brought about by the COVID-19 pandemic [39]. Documented challenges also encompassed the task of controlling patients’ adherence to preventative regulations, coupled with encountering verbal aggression from patients who were skeptical of COVID-19 [41].

#### Organizational level

At the organizational level, inadequate facility infrastructure and resources posed significant obstacles to the effective enactment of PHC nurses’ roles during the pandemic. These barriers included a lack of personal protective equipment [40, 41, 48], which further exacerbated the sense of insecurity [40]. Shortages of medications and contraceptives as well as limited access to water and sanitation facilities were also identified as barriers [41]. Furthermore, the scarcity of diagnostic resources and healthcare professionals within PHC settings, which were pre-existing concerns before the pandemic, further exacerbated the challenges faced by PHC nurses during the current pandemic [45]. In a qualitative study of seven PHC nurses in Japan [39], nurses often encountered situations in which they felt compelled to deliver in-person care without sufficient preventive measures.

PHC nurses encounter challenges arising from a lack of clear guidelines, notably concerning the absence of clear protocols regarding the proper utilization of personal protective equipment and consistent accessibility of up-to-date COVID-19-related information [38, 40]. Moreover, they experienced inadequate supervision and support, particularly concerning staff training on COVID-19 prevention and control measures [36, 48]. In a survey examining the preparedness of PHC nurses for COVID-19 in South Africa [36], over half of respondents indicated that they either did not receive training or lacked confidence in the training they received regarding preventive and control measures.

The increased workload and subsequent worsening of patient care have been identified as challenges [18, 37, 45, 48]. Three studies reported that participants perceived the quality of PHC services to have worsened or to have been adversely affected by the increased workload owing to the time allocated to triage and screening, and additional nursing responsibilities [18, 37, 48].

While PHC nurses perceived telehealth as an effective tool for enabling patients to access care during the pandemic, they also identified it as a barrier to patient care [38, 44, 48]. A qualitative study examining PHC nurses’ experiences using telehealth during the pandemic in Australia reported mixed results regarding preparedness for the rapid implementation of telehealth [44]. While telehealth has been rapidly embraced and accepted in PHC settings, where it is already being used or where appropriate resources are available, in environments with inadequate equipment or insufficient staff capabilities, PHC nurses perceived its implementation as not fully established [44]. While PHC nurses acknowledged the efficiency of telehealth [38, 44, 47, 48], they also considered its utilization as a barrier to complex clinical assessments, especially for non-COVID-19 health conditions [44, 48]. It has the potential to undermine holistic care by restricting non-verbal cues and overall impressions, discontinuing home visits for homebound patients, and diminishing access to vulnerable individuals [48]. The lack of adequate funding for telehealth provided by PHC nurses posed an additional challenge that impeded patient care and nurse retention [38, 44].

#### Community- and societal-level

Community- and societal-level challenges referred to by PHC nurses have been reported in seven studies [38–41, 45]. For instance, the lack of governmental education and guidelines was challenging [38, 40]. In a qualitative study of school nurses’ experiences during the COVID-19 pandemic in Hong Kong, conflicting and unclear messages from the government induced initial stress, feelings of uncertainty, and anxiety in nurses [40]. Insufficient availability of COVID-19 education for the general public has been identified as a hindrance to providing high-quality care [38]. The predominant emphasis on epidemiological surveillance and medical-hospital care, coupled with the ambiguous scope of PHC and the lack of workforce and resources for PHC, posed additional obstacles to the effective implementation of PHC nurses’ roles [45]. Furthermore, PHC nurses perceived low societal morale [41] and concerns regarding security measures implemented by prefectural and national governments as additional challenges [39].

The limitations imposed by the funding models of health systems posed a significant obstacle to the effective fulfillment of nursing roles in PHC [38, 41, 44]. The absence of incentives offered at the government level was also identified as a challenge to the delivery of PHC [41]. Additionally, while nurses have proactively incorporated telehealth in response to the pandemic, the noticeable challenge of sustaining nurse-led or nurse-involved telehealth efforts stemmed from a lack of government support in terms of rebates for telehealth services [38, 44]. A qualitative study exploring the experiences of PHC nurses in using telehealth during the pandemic revealed that the initial provision of government funding for telehealth exclusively to physicians and allied health professionals, along with the delayed provision of funding for telehealth services provided by general practice nurses, had repercussions on nurses’ job security [44].

### Strategies used to overcome challenges

Facilitators and strategies, both implemented and suggested for the effective implementation of nursing roles in PHC, were organized according to the socioecological framework. At the individual level, addressing nurses’ needs to engage in self-care to enhance well-being, including interventions for managing stress and facilitating access to both informally and formally funded mental health services, were identified as areas for improvement [36, 38, 48].

At the interpersonal level, providing support for personal caregiving responsibilities, encompassing enhancements in homeschooling and childcare systems, is also acknowledged as a crucial area that requires improvement [36]. Peer support offered in-person and online has emerged as a strategy to mitigate challenges in implementing nursing roles [40, 48]. In a qualitative study involving school nurses in Hong Kong, establishing connections with fellow school nurses proved pivotal for information-sharing through professional peer support. Leveraging social media platforms facilitated informal communication among nurses [40]. Solidarity among PHC professionals was also acknowledged as a supportive factor in addressing challenges during the pandemic [45].

At the organizational level, effective communication between team members and patients has been recognized as a strategy to alleviate challenges and create a safe environment during the pandemic [38, 40, 45, 48]. In a qualitative study centered on PHC care for older adults provided by nurses in Thailand during the pandemic, the utilization of customized teaching materials specifically tailored to this demographic, alongside the use of straightforward and comprehensible language and dialects, effectively delivered older-adult-targeted health promotion education [45]. Adopting technologies such as virtual meetings and telehealth played a pivotal role in enhancing the implementation of nurses’ roles by significantly saving time and improving efficiency [44, 47, 48]. The need for reliable technology provision and staff training programs was also recognized as an area for improvement [48].

At the community and societal levels, the growing recognition of the value and significant contributions made by nurses during the pandemic served as catalysts for fostering resilience and proactivity in nurses’ role implementation [38, 40]. Government subsidies for nurses to provide telehealth services were also identified as vital for retaining nursing professionals and enabling the continual delivery of high-quality care throughout the pandemic [38, 44]. Finally, establishing robust networks involving various organizations, including health-related agencies, logical politicians, and the private sector, was a significant facilitator in ensuring effective community surveillance and COVID-19 prevention and control [45].

## Discussion

To the best of our knowledge, this scoping review is one of the first comprehensive studies using the socioecological model of human development to depict nursing roles and experiences in PHC settings during the COVID-19 pandemic. Amid the challenges posed by the pandemic, PHC nurses have proactively extended their roles to address the COVID-19 crisis effectively. In addition to surveillance activities mandated by governmental and organizational bodies, nurses have become role models by supporting patients and colleagues in adhering to preventive measures while also empowering community volunteers to enhance their capabilities. Furthermore, nurses actively participated in initiatives to alleviate COVID-19-related social stigma and collaborated with diverse organizations to improve the care continuity of COVID-19 patients and ensure efficient surveillance. Nurses also navigated the influx of information, ensuring they were updated with the most recent research and trends. They were pivotal in shaping organizational protocols. They have also embraced digital technologies to adapt and enhance service-delivery models, demonstrating flexibility and innovation.

Echoing a previous scoping review [49], PHC nurses assumed a pivotal role in primary care and the broader realm of public health, fostering integrated care between these two domains. An integrative approach is crucial to enhance healthcare access for all, especially in social disasters such as novel infectious disease pandemics. Tailoring services to the needs of vulnerable populations and facilitating informed resource allocation across clinical settings and communities is essential [50]. To enhance integrated healthcare service delivery, government entities, health authorities, and healthcare organizations need to recognize and integrate PHC nurses’ expanding and emerging roles within the scope of practice, ensuring their effective implementation while avoiding excessive regulatory constraints. Additionally, essential professional development programs enable nurses to build competencies in these evolving roles. Thus, they are crucial in preparing PHC nurses for future pandemics.

The socioecological framework posits that multiple nested levels influence human development and experiences within a structured system, including intrapersonal, interpersonal, institutional, and societal factors [34, 35]. This framework helps conceptualize the complex interplay between nurses and their environments during the COVID-19 pandemic, the multiple levels of barriers and facilitators, and the multidimensional experiences of PHC nurses [51]. Our review revealed that factors at different levels within the socioecological framework influence the challenges experienced by nurses. For example, a sense of insecurity at the individual level was exacerbated by job insecurity and a shortage of accessible resources, including personal protective equipment, at the organizational level. Acknowledging the importance of PHC nurses and their contributions during the pandemic at the social level fostered their individual resilience and proactivity.

Healthcare professionals’ resilience relates to managing the challenges arising from significant adversity with external support without compromising the quality of care, health, and well-being [52]. Despite the scant evidence, this review also highlighted the resilience of PHC nurses during the pandemic. Feelings of helplessness and anxiety initially arose because of the perceived vulnerability to infection, shortage of personal protective equipment, and job insecurity. However, these emotions transformed into adaptability and a growing sense of control [39, 40, 47]. Recognition of their roles and contributions, and a strong sense of professionalism, have emerged as significant in fostering resilience [39]. A prior qualitative study on healthcare workers’ resilience in India also found that local and community recognition, as well as support from their networks and engagement in individual self-care activities such as faith-based activities, played significant roles in fostering the resilience of PHC providers [53]. In line with our results, another systematic review revealed the multifaceted nature of healthcare professionals’ resilience by incorporating personal, social, and workplace features [52]. Building a strong infrastructure to support nurse self-care needs, providing continuing professional development initiatives, and securing official recognition from the government helped to enhance their resilience [53].

The challenges presented by the global pandemic emphasized the vital and irreplaceable role of digital technologies [54]. As noted in this review, nurses acknowledged that telehealth was essential to PHC during the pandemic, enabling effective strategies and ensuring the safety of patients, healthcare professionals, and other stakeholders [55]. However, the rapid implementation of telehealth without adequate resources and capabilities resulted in a lack of confidence and readiness among nurses. Additionally, although telehealth broadened access to healthcare services for vulnerable individuals [56], this review highlighted that evidence-based guidelines tailored to specific target users are essential for optimizing PHC services to ensure they have easy access to digital technologies. Governmental and international investments should prioritize building and improving technology infrastructure and implementing digital literacy education initiatives targeting end users, including patients, caregivers, and nurses, especially in underserved low- and middle-income countries [57]. Finally, following Hoffman’s [58] report highlighting reimbursement as a substantial obstacle to telehealth adoption, this review underscored deficient reimbursement and inadequate funding for nurse-led and nurse-engaged telehealth initiatives in PHC settings. Therefore, legal and regulatory measures regarding payments for virtual PHC services delivered by nurses are imperative.

Healthcare crises that occurred during previous epidemics and pandemics challenged healthcare systems and exposed their systematic weaknesses; however, they also presented valuable lessons that drove innovation and reform. For example, Taiwan learned from its failure to respond to the 2002 SARS outbreak, and during the COVID-19 pandemic, the critical role of nurses, along with the Taiwanese government’s response, was recognized as a model by many other countries [59]. During the SARS outbreak in Taiwan, media sensationalism not only heightened public anxiety but also caused psychological stress among nurses, which led to a nursing shortage [60]. Learning from the SARS situation, the government and nursing organizations jointly prioritized professional and legal protections of nurses, ensuring adequate personal protective equipment, appropriate nursing staffing, sufficient rest to ensure nurses’ physical and psychological resilience, and providing subsidies as necessary to nurses [59]. An online platform for nurses to file complaints was established, and educational materials for both nurses and the public were developed and distributed via online platforms and social media. This helped nurses prepare to cope with COVID-19, resulting in fewer resignations among nurses compared to the SARS epidemic [59]. At the beginning of the COVID-19 pandemic, a nurse-led quarantine care call center was also established in Taiwan, effectively responding to the crisis and highlighting the pivotal role of nurses in epidemic and pandemic situations [61].

PHC must be delivered efficiently, treating illness and maintaining health, using the most effective approaches to meet the specific needs of diverse populations and underserved populations, and ensuring a health system that connects and links beneficiaries to services across sectors [62]. What we have learned from the COVID-19 pandemic is that nursing can play a transformative role in driving this transformation of primary care. Nursing care that uses and is based on innovative nursing technologies can play a more comprehensive role in strengthening the above systems.

### Strengths and limitations

This scoping review has several strengths. The review of both quantitative and qualitative studies enabled a comparison of the findings and the provision of detailed contextual information. By organizing the findings based on the socioecological model, different dimensions of PHC nurses’ experiences were systematically examined. A major limitation of this scoping review was the wide range of geographical locations where the included studies were conducted. This diversity posed a challenge in effectively comparing studies while considering the complete array of pertinent policies, regulations, and healthcare environments regarding the scope of practice for PHC nurses in each country. Although we only reviewed peer-reviewed studies, we acknowledge the potential existence of alternative sources, such as government reports. Finally, given that the search was limited to publications in English, findings from non-English articles are unknown.

## Conclusion

While the COVID-19 pandemic has considerably affected the healthcare system, the challenges imposed by the pandemic highlighted the vital and indispensable role of PHC nurses. Nurses proactively embraced the emerging role of the pandemic in addressing the related crisis. The challenges imposed by the pandemic were multilevel issues arising from specific individual, interpersonal, organizational, and societal circumstances. Classifying barriers and challenges, as well as facilitators and strategies, using a socioecological framework provides organizational leadership and policymakers with new insights to develop multilevel interventions. These interventions can effectively address the support needs of PHC nurses through a comprehensive system-based approach. The findings also have policy implications for addressing the support needs of PHC nurses at the individual, interpersonal, organizational, and societal levels with the overarching goal of enhancing the overall quality of the PHC system.

## Electronic supplementary material

Below is the link to the electronic supplementary material.

Supplementary Material 1

Supplementary Material 2

## Data Availability

The data including detailed descriptions of the literature and search outcome are provided within the manuscript and electronic supplementary information file.

## Abbreviations

COVID-19: Coronavirus disease
OECD: Organization for Economic Co-operation and Development
PHC: Primary health care
PRISMA-ScR: Preferred Reporting Items for Systematic Reviews and Meta-Analysis Extension for Scoping Reviews

## References

[1] Bell L, van Gemert C, Merilles OE Jr., Cash HL, Stoové M, Hellard M. The impact of COVID-19 on public health systems in the Pacific Island Countries and territories. Lancet Reg Health West Pac. 2022;25:100498. 10.1016/j.lanwpc.2022.100498 35785109 10.1016/j.lanwpc.2022.100498 PMC9230438

[2] Maravilla J, Catiwa J, Guariño R, Yap JF, Pagatpatan C Jr., Orolfo DD, et al. Exploring indirect impacts of COVID-19 on local health systems from the perspectives of health workers and higher education stakeholders in the Philippines using a phenomenological approach. Lancet Reg Health West Pac. 2023;30:100585. 10.1016/j.lanwpc.2022.100585 36128337 10.1016/j.lanwpc.2022.100585 PMC9477542

[3] Organization for Economic Co-operation and Development (OECD). Strengthening the frontline: how primary health care helps health systems adapt during the COVID-19 pandemic. Paris: OECD Publishing; 2021. 10.1787/9a5ae6da-en

[4] de Meijer C, Wouterse B, Polder J, Koopmanschap M. The effect of population aging on health expenditure growth: a critical review. Eur J Ageing. 2013;10:353–61. 10.1007/s10433-013-0280-x 28804308 10.1007/s10433-013-0280-x PMC5549212

[5] Wouterse B, Huisman M, Meijboom BR, Deeg DJH, Polder JJ. The effect of trends in health and longevity on health services use by older adults. BMC Health Serv Res. 2015;15:574. 10.1186/s12913-015-1239-8 26704342 10.1186/s12913-015-1239-8 PMC4690430

[6] Bollyky TJ, Templin T, Cohen M, Dieleman JL. Lower-income countries that face the most rapid shift in noncommunicable disease burden are also the least prepared. Health Aff (Millwood). 2017;36:1866–75. 10.1377/hlthaff.2017.0708 29137514 10.1377/hlthaff.2017.0708 PMC7705176

[7] Liu L, Villavicencio F, Yeung D, Perin J, Lopez G, Strong KL, et al. National, regional, and global causes of mortality in 5–19-year-olds from 2000 to 2019: a systematic analysis. Lancet Glob Health. 2022;10:e337–47. 10.1016/S2214-109X(21)00566-0 35180417 10.1016/S2214-109X(21)00566-0 PMC8864304

[8] Roser M, Ritchie H, Spooner F. Burden of disease: how is the burden of disease distributed and how did it change over time? 2021. https://ourworldindata.org/burden-of-disease. 15 Feb 2024.

[9] Kämpfen F, Wijemunige N, Evangelista B, Aging. Non-communicable diseases, and old-age disability in low- and middle-income countries: a challenge for global health. Int J Public Health. 2018;63:1011–2. 10.1007/s00038-018-1137-z 29951746 10.1007/s00038-018-1137-z

[10] Baker RE, Mahmud AS, Miller IF, Rajeev M, Rasambainarivo F, Rice BL, et al. Infectious disease in an era of global change. Nat Rev Microbiol. 2022;20:193–205. 10.1038/s41579-021-00639-z 34646006 10.1038/s41579-021-00639-z PMC8513385

[11] Bernacki K, Keister A, Sapiro N, Joo JS, Mattle L. Impact of COVID-19 on patient and healthcare professional attitudes, beliefs, and behaviors toward the healthcare system and on the dynamics of the healthcare pathway. BMC Health Serv Res. 2021;21:1309. 10.1186/s12913-021-07237-y 34872537 10.1186/s12913-021-07237-y PMC8646017

[12] World Health Organization, United Nations Children’s Fund (UNICEF). A vision for primary health care in the 21st century: towards universal health coverage and the sustainable development goals. CC NC-SA 3.0 IGO. 2018. https://iris.who.int/handle/10665/328065. 15 Aug 2023.

[13] World Health Organization. Declaration of Astana Global conference on primary health care. 2018. https://www.who.int/publications/i/item/WHO-HIS-SDS-2018.61. 15 Aug 2023.

[14] Hanson K, Brikci N, Erlangga D, Alebachew A, De Allegri M, Balabanova D, et al. The Lancet Global Health Commission on financing primary health care: putting people at the centre. Lancet Glob Health. 2022;10:e715–72. 10.1016/S2214-109X(22)00005-5 35390342 10.1016/S2214-109X(22)00005-5 PMC9005653

[15] WHO Regional Office for the Western Pacific. Role of primary care in the COVID-19 response. 2020. https://www.who.int/publications/i/item/WPR-DSE-2020-004. 15 Aug 2023.

[16] World Health Organization. State of the world’s nursing 2020: investing in education, jobs and leadership. 2020. https://apps.who.int/iris/handle/10665/331677. 15 Aug 2023.

[17] Jackson D, Bradbury-Jones C, Baptiste D, Gelling L, Morin K, Neville S, et al. Life in the pandemic: some reflections on nursing in the context of COVID-19. J Clin Nurs. 2020;29:2041–3. 10.1111/jocn.15257 32281185 10.1111/jocn.15257 PMC7228254

[18] Halcomb E, McInnes S, Williams A, Ashley C, James S, Fernandez R, et al. The experiences of primary healthcare nurses during the COVID-19 pandemic in Australia. J Nurs Scholarsh. 2020;52:553–63. 10.1111/jnu.12589 32735758 10.1111/jnu.12589 PMC7436753

[19] Brault I, Kilpatrick K, D’Amour D, Contandriopoulos D, Chouinard V, Dubois CA, et al. Role clarification processes for better integration of nurse practitioners into primary healthcare teams: a multiple-case study. Nurs Res Pract. 2014;2014:170514. 10.1155/2014/170514 25692039 10.1155/2014/170514 PMC4322308

[20] Busca E, Savatteri A, Calafato TL, Mazzoleni B, Barisone M, Dal Molin A. Barriers and facilitators to the implementation of nurse’s role in primary care settings: an integrative review. BMC Nurs. 2021;20:171. 10.1186/s12912-021-00696-y 34530813 10.1186/s12912-021-00696-y PMC8444166

[21] Kumpunen S, Webb E, Permanand G, Zheleznyakov E, Edwards N, van Ginneken E, et al. Transformations in the landscape of primary health care during COVID-19: themes from the European region. Health Policy. 2022;126:391–7. 10.1016/j.healthpol.2021.08.002 34489126 10.1016/j.healthpol.2021.08.002 PMC8364142

[22] Al-Rawashdeh N, Al Bashtawy M, Ozaybi N, Alkhawaldeh A, Albashtawy B, Albashtawy S et al. Nurses roles in providing care for patient with COVID-19. EC Emerg Med Crit Care. 2020;5.

[23] World Health Organization. Primary health care measurement framework and indicators: monitoring health systems through a primary health care lens. 2022. https://iris.who.int/bitstream/handle/10665/352205/9789240044210-eng.pdf?sequence=1. 15 Aug 2023.

[24] Aromataris E, Munn Z, editors. JBI Manual for evidence synthesis. Adelaide: JBI; 2020. https://synthesismanual.jbi.global. 10.46658/JBIMES-20-01

[25] Peters MDJ, Marnie C, Tricco AC, Pollock D, Munn Z, Alexander L, et al. Updated methodological guidance for the conduct of scoping reviews. JBI Evid Synth. 2020;18:2119–26. 10.11124/JBIES-20-00167 33038124 10.11124/JBIES-20-00167

[26] Tricco AC, Lillie E, Zarin W, O’Brien KK, Colquhoun H, Levac D, et al. PRISMA extension for scoping reviews (PRISMA-ScR): checklist and explanation. Ann Intern Med. 2018;169:467–73. 10.7326/M18-0850 30178033 10.7326/M18-0850

[27] World Health Organization. WHO Director-General’s opening remarks at the media briefing on COVID-19–11 March 2020. 2020. https://www.who.int/director-general/speeches/detail/who-director-general-s-opening-remarks-at-the-media-briefing-on-covid-19---11-march-2020. 14 Sep 2024.

[28] Higgins JP, Thomas J, Chandler J, Cumpston M, Li T, Page MJ, et al. Cochrane handbook for systematic reviews of interventions. New Jersey: Wiley; 2019. 10.1002/14651858.ED000142 PMC10284251 31643080

[29] Godin K, Stapleton J, Kirkpatrick SI, Hanning RM, Leatherdale ST. Applying systematic review search methods to the grey literature: a case study examining guidelines for school-based breakfast programs in Canada. Syst Rev. 2015;4:138. 10.1186/s13643-015-0125-0 26494010 10.1186/s13643-015-0125-0 PMC4619264

[30] Mathews M, Spencer S, Hedden L, Marshall EG, Lukewich J, Meredith L, et al. Development of a primary care pandemic plan informed by in-depth policy analysis and interviews with family physicians across Canada during COVID-19: a qualitative case study protocol. BMJ Open. 2021;11:e048209. 10.1136/bmjopen-2020-048209 10.1136/bmjopen-2020-048209 PMC8300554 34301660

[31] Woo BFY, Tam WWS, Williams MY, Ow Yong JQY, Cheong Z, Ong YC, et al. Characteristics, methodological, and reporting quality of scoping reviews published in nursing journals: a systematic review. J Nurs Scholarsh. 2023;5:874–85. 10.1111/jnu.12861 10.1111/jnu.12861 36494752

[32] Hong QN, Fàbregues S, Bartlett G, Boardman F, Cargo M, Dagenais P, et al. The mixed methods appraisal tool (MMAT) version 2018 for information professionals and researchers. Educ Inf. 2018;34(4):285–91.

[33] Garrard J. Health sciences literature review made easy. Massachusetts: Jones & Bartlett Publishers Learning; 2020.

[34] Bronfenbrenner U, Morris PA. The bioecological model of human development. In: William ED, editor. Handbook of child psychology. 6th ed. New Jersey: Wiley; 2006. pp. 793–828.

[35] Bronfenbrenner U. Toward an experimental ecology of human development. Am Psychol. 1977;32:513–31. 10.1037/0003-066X.32.7.513

[36] Crowley T, Kitshoff D, De Lange-Cloete F, Baron J, De Lange S, Young C, et al. Primary care nurses’ preparedness for COVID-19 in the Western Cape Province, South Africa. Afr J Prim Health Care Fam Med. 2021;13:e1–8. 10.4102/phcfm.v13i1.2879 10.4102/phcfm.v13i1.2879 PMC8182487 34082553

[37] Crowley T, Kitshoff D, De Lange-Cloete F, Baron J, De Lange S, Young C, et al. Reorganisation of primary care services during Covid-19 in the Western Cape, South Africa: perspectives of primary care nurses. S Afr Fam Pract (2004). 2021;63(e1–10). 10.4102/safp.v63i1.5358 10.4102/safp.v63i1.5358 PMC8661113 34879690

[38] Halcomb E, Williams A, Ashley C, McInnes S, Stephen C, Calma K, et al. The support needs of Australian primary health care nurses during the COVID-19 pandemic. J Nurs Manag. 2020;28:1553–60. 10.1111/jonm.13108 32713047 10.1111/jonm.13108

[39] Mizumoto J, Mitsuyama T, Kumagaya S, Eto M, Izumiya M, Horita S. Primary care nurses during the coronavirus disaster and their struggle: qualitative research. J Gen Fam Med. 2022;23:343–50. 10.1002/jgf2.566 36093220 10.1002/jgf2.566 PMC9444012

[40] Lee RLT, West S, Tang ACY, Cheng HY, Chong CYY, Chien WT, et al. A qualitative exploration of the experiences of school nurses during COVID-19 pandemic as the frontline primary health care professionals. Nurs Outlook. 2021;69:399–408. 10.1016/j.outlook.2020.12.003 33541726 10.1016/j.outlook.2020.12.003 PMC7834307

[41] Adelekan B, Goldson E, Abubakar Z, Mueller U, Alayande A, Ojogun T, et al. Effect of COVID-19 pandemic on provision of sexual and reproductive health services in primary health facilities in Nigeria: a cross-sectional study. Reprod Health. 2021;18:166. 10.1186/s12978-021-01217-5 34348757 10.1186/s12978-021-01217-5 PMC8334336

[42] James S, Ashley C, Williams A, Desborough J, McInnes S, Calma K, et al. Experiences of Australian primary healthcare nurses in using telehealth during COVID-19: a qualitative study. BMJ Open. 2021;11:e049095. 10.1136/bmjopen-2021-049095 10.1136/bmjopen-2021-049095 PMC8350972 34362804

[43] Martins ALX, David HMSL, Koopmans FF, Martínez-Riera JR. Crisis, work and nursing: an ethnographic narrative of the coronavirus pandemic in primary care in Spain. Rev Bras Enferm. 2021;75(Suppl 1):e20210069. 10.1590/0034-7167-2021-0069 10.1590/0034-7167-2021-0069 34669788

[44] Halcomb E, Fernandez R, Ashley C, McInnes S, Stephen C, Calma K, et al. The impact of COVID-19 on primary health care delivery in Australia. J Adv Nurs. 2022;78:1327–36. 10.1111/jan.15046 34554594 10.1111/jan.15046

[45] Akbar MA, Juniarti N, Yamin A. The roles of community health nurses’ in Covid-19 management in Indonesia: a qualitative study. Int J Community Based Nurs Midwifery. 2022;10:96–109. 10.30476/IJCBNM.2021.90884.1739 35372635 10.30476/IJCBNM.2021.90884.1739 PMC8957658

[46] Yodsuban P, Pengpid S, Amornchai R, Siripoon P, Kasemsuk W, Buasai N. The roles of community health nurses for older adults during the COVID-19 pandemic in Northeastern Thailand: a qualitative study. Int J Nurs Sci. 2023;10:53–63. 10.1016/j.ijnss.2022.12.014 36590312 10.1016/j.ijnss.2022.12.014 PMC9792194

[47] Nilsen P, Fernemark H, Seing I, Schildmeijer K, Skagerström J. Seven lessons from the coronavirus pandemic for primary health care: a qualitative study of registered and assistant nurses in Sweden. Scand J Caring Sci. 2022;36:1197–205. 10.1111/scs.13082 35466416 10.1111/scs.13082 PMC9115448

[48] Russell A, De Wildt G, Grut M, Greenfield S, Clarke J. What can general practice learn from primary care nurses’ and healthcare assistants’ experiences of the COVID-19 pandemic? A qualitative study. BMJ Open. 2022;12:e055955. 10.1136/bmjopen-2021-055955 10.1136/bmjopen-2021-055955 PMC8927928 35292497

[49] Swanson M, Wong ST, Martin-Misener R, Browne AJ. The role of registered nurses in primary care and public health collaboration: a scoping review. Nurs Open. 2020;7:1197–207. 10.1002/nop2.496 32587740 10.1002/nop2.496 PMC7308712

[50] Sathian B, van Teijlingen E, Simkhada P, Editorial. Integrated health service delivery and COVID-19. Front Public Health. 2022;10:1008777. 10.3389/fpubh.2022.1008777 36187604 10.3389/fpubh.2022.1008777 PMC9521494

[51] Chemali S, Mari-Sáez A, El Bcheraoui C, Weishaar H. Health care workers’ experiences during the COVID-19 pandemic: a scoping review. Hum Resour Health. 2022;20:27. 10.1186/s12960-022-00724-1 35331261 10.1186/s12960-022-00724-1 PMC8943506

[52] Robertson HD, Elliott AM, Burton C, Iversen L, Murchie P, Porteous T, et al. Resilience of primary healthcare professionals: a systematic review. Br J Gen Pract. 2016;66:e423–33. 10.3399/bjgp16X685261 27162208 10.3399/bjgp16X685261 PMC4871308

[53] Golechha M, Bohra T, Patel M, Khetrapal S. Healthcare worker resilience during the COVID-19 pandemic: a qualitative study of primary care providers in India. World Med Health Policy. 2022;14:6–18. 10.1002/wmh3.483 34909242 10.1002/wmh3.483 PMC8662083

[54] Whitelaw S, Mamas MA, Topol E, Van Spall HGC. Applications of digital technology in COVID-19 pandemic planning and response. Lancet Digit Health. 2020;2:e435–40. 10.1016/S2589-7500(20)30142-4 32835201 10.1016/S2589-7500(20)30142-4 PMC7324092

[55] Monaghesh E, Hajizadeh A. The role of telehealth during COVID-19 outbreak: a systematic review based on current evidence. BMC Public Health. 2020;20:1193. 10.1186/s12889-020-09301-4 32738884 10.1186/s12889-020-09301-4 PMC7395209

[56] Shah DA, Sall D, Peng W, Sharer R, Essary AC, Radhakrishnan P. Exploring the role of telehealth in providing equitable healthcare to the vulnerable patient population during COVID-19. J Telemed Telecare. 2022;11:1357633X221113711. 10.1177/1357633X221113711 10.1177/1357633X221113711 PMC9283958 35833345

[57] Eslami Jahromi M, Ayatollahi H. Utilization of telehealth to manage the Covid-19 pandemic in low- and middle-income countries: a scoping review. J Am Med Inf Assoc. 2023;30:738–51. 10.1093/jamia/ocac250 10.1093/jamia/ocac250 PMC10018263 36565464

[58] Hoffman DA. Increasing access to care: telehealth during COVID-19. J Law Biosci. 2020;7:lsaa043. 10.1093/jlb/lsaa043 32843985 10.1093/jlb/lsaa043 PMC7337821

[59] Tsay SF, Kao CC, Wang HH, Lin CC. Nursing’s response to COVID-19: lessons learned from SARS in Taiwan. Int J Nurs Stud. 2020;108:103587. 32388221 10.1016/j.ijnurstu.2020.103587 PMC7151482

[60] Wu YC. Standardized operation process for communication with mass media during disease outbreaks: based on experiences from SARS. Taiwan J Public Health. 2007;26:241–9.

[61] Huang LH, Chen CM, Chen SF, Wang HH. Roles of nurses and national nurses associations in combating COVID-19: Taiwan experience. Int Nurs Rev. 2020;67(3):318–22. 32761608 10.1111/inr.12609 PMC7436573

[62] WHO Regional Office for the Western Pacific. Regional framework on the future of primary health care in the western Pacific. 2023. https://www.who.int/publications/i/item/9789290620129. 15 Aug 2023.

