# Supplementary material for: Roles and experiences of nurses in primary health care during the COVID-19 pandemic: a scoping review
Source: BMC Nurs. 2024 Oct 11;23:740. doi: 10.1186/s12912-024-02406-w (PMC11468121; doi:10.1186/s12912-024-02406-w)
Supplement: Supplementary file 1 — Supplementary Material 1 [file 12912_2024_2406_MOESM1_ESM.docx]

**Appendix A**

Supplementary Table 1. Search strategy

Search date: June 30, 2023

| Database | Nurse | COVID-19 pandemic | Primary health care setting |
| --- | --- | --- | --- |
| PubMed | "Nurse"[Title/Abstract] OR "Nurses"[Title/Abstract] OR "Nursing"[Title/Abstract] OR "Nurse-Led"[Title/Abstract] OR "Nurses"[MeSH Terms] OR "Nursing"[MeSH Terms] | "COVID-19"[Title/Abstract] OR "COVID-19 Vaccines"[Title/Abstract] OR "COVID-19 Testing"[Title/Abstract] OR "Severe Acute Respiratory Syndrome"[Title/Abstract] OR "Coronavirus"[Title/Abstract] OR "SARS-CoV-2"[Title/Abstract] OR "Post-Acute COVID-19 syndrome"[Title/Abstract] OR "NCOV"[Title/Abstract] OR "COVID-19 Pandemic"[Title/Abstract] OR "COVID-19"[MeSH Terms] OR "COVID-19 Vaccines"[MeSH Terms] OR "COVID-19 testing"[MeSH Terms] OR "Coronavirus"[MeSH Terms] | "Primary care"[Title/Abstract] OR "Primary Healthcare"[Title/Abstract] OR "Primary Health Care" [MeSH Terms] |
| CINAHL | TI Nurse OR AB Nurse OR TI Nurses OR AB Nurses OR TI Nursing OR AB Nursing OR TI Nurse-Led OR AB Nurse-Led OR (MH "Nurses+") | TI COVID-19 OR AB COVID-19 OR TI COVID-19 Vaccines OR AB COVID-19 Vaccines OR TI COVID-19 Testing OR AB COVID-19 Testing OR TI Severe Acute Respiratory Syndrome OR AB Severe Acute Respiratory Syndrome OR TI Coronavirus OR AB Coronavirus TI SARS-CoV-2 OR AB SARS-CoV-2 OR Post-Acute COVID-19 syndrome OR AB Post-Acute COVID-19 syndrome OR NCOV OR AB NCOV OR TI COVID-19 Pandemic OR AB COVID-19 Pandemic OR (MH "COVID-19") OR (MH "COVID-19 Testing") OR (MH "COVID-19 Vaccines") OR (MH "SARS-CoV-2") OR (MH "Post-Acute COVID-19 Syndrome") OR (MH "COVID-19 Pandemic") | TI Primary care OR AB Primary care OR TI Primary Healthcare OR AB Primary Healthcare OR (MH "Primary Health Care") |
| EMBASE | Nurse:ab,ti OR Nurses:ab,ti OR Nursing:ab,ti OR Nurse-Led:ab,ti OR 'nurse':exp OR 'nursing':exp | COVID-19:ab,ti OR COVID-19 Vaccines:ab,ti OR COVID-19 Testing:ab,ti OR Severe Acute Respiratory Syndrome:ab,ti OR Coronavirus:ab,ti OR SARS-CoV-2:ab,ti OR Post-Acute COVID-19 syndrome:ab,ti OR NCOV:ab,ti OR COVID-19 Pandemic:ab,ti OR 'coronavirus disease 2019':exp OR 'Severe acute respiratory syndrome coronavirus 2':exp OR 'coronavirus disease 2019':exp | Primary care:ab,ti OR Primary Healthcare:ab,ti OR 'primary health care':exp |
| PsycINFO | ab(Nurse) OR ti(Nurse) OR ab(Nurses) OR ti(Nurses) OR ab(Nursing) OR ti(Nursing) OR ab(nurse-led) OR ti(Nurse led) OR MAINSUBJECT.EXACT("Nurses") OR MAINSUBJECT.EXACT("Nursing") | ab(COVID-19) OR ti(COVID-19) OR ab(COVID-19 Vaccines) OR ti(COVID-19 Vaccines) OR ab(COVID-19 Testing) OR ti(COVID-19 Testing) OR ab(Severe Acute Respiratory Syndrome) OR ti(Severe Acute Respiratory Syndrome) OR ab(Coronavirus) OR ti(Coronavirus) OR ab(SARS-CoV-2) OR ti(SARS-CoV-2) OR ab(Post-Acute COVID-19 syndrome) OR ti(Post-Acute COVID-19 syndrome) OR ab(NCOV) OR ti(NCOV) OR ab(COVID-19 Pandemic) OR ti(COVID-19 Pandemic) OR MAINSUBJECT.EXACT.EXPLODE("COVID-19") OR MAINSUBJECT.EXACT.EXPLODE("COVID-19 Testing") | ab(Primary care) OR ti(Primary care) OR ab(Primary Healthcare) OR ti(Primary Healthcare) OR MAINSUBJECT.EXACT.EXPLODE("Primary Health Care") |
| SCOPUS | ( TITLE ( ( "Nurse" OR "Nurses" OR "Nursing" OR "Nurse-Led" ) ) OR ABS ( ( "Nurse" OR "Nurses" OR "Nursing" OR "Nurse-Led" ) ) ) | ( TITLE ( ("COVID-19" OR "COVID-19 Vaccines" OR "COVID-19 Testing" OR "Severe Acute Respiratory Syndrome" OR "Coronavirus" OR "SARS-CoV-2" OR "Post-Acute COVID-19 syndrome" OR "NCOV" OR "COVID-19 Pandemic") ) OR ABS ( ( "COVID-19" OR "COVID-19 Vaccines" OR "COVID-19 Testing" OR "Severe Acute Respiratory Syndrome" OR "Coronavirus" OR "SARS-CoV-2" OR "Post-Acute COVID-19 syndrome" OR "NCOV" OR "COVID-19 Pandemic") ) ) | TITLE ( ( "Primary care" OR "Primary Healthcare" ) ) OR ABS ( ( "Primary care" OR "Primary Healthcare" ) ) ) |
