# Supplementary material for: Roles and experiences of nurses in primary health care during the COVID-19 pandemic: a scoping review
Source: BMC Nurs. 2024 Oct 11;23:740. doi: 10.1186/s12912-024-02406-w (PMC11468121; doi:10.1186/s12912-024-02406-w)
Supplement: Supplementary file 2 — Supplementary Material 2 [file 12912_2024_2406_MOESM2_ESM.docx]

**Appendix B**

Supplementary Table 1. Critical Appraisal Results Using Mixed Methods Appraisal Tool (MMAT) Version 2018 for Quantitative Descriptive Studies

| Author  (Year) | Is the sample strategy relevant to address the research question? | Is the sample representative of the target population? | Are the measurements appropriate? | Is the risk of nonresponse bias low? | Is the statistical analysis appropriate to answer the research question? | Limitations |
| --- | --- | --- | --- | --- | --- | --- |
| Adelekan et al. (2021) | No | No | Yes | Yes | Yes | - Potential for selection bias and recall bias due to only one key informant (a head nurse/midwife) per a facility interviewed. |
| Crowley et al. (2021) | No | No | Yes | No | Yes | - Convenience sampling from one university, potential selection bias and limited generalizability. - Low response rate. |
| Crowley et al. (2021) | No | No | Yes | No | Yes | - Convenience sampling from one university, potential selection bias, and limited generalizability. - Low response rate. |
| Halcomb et al. (2022) | Yes | Yes | Yes | Can’t tell | Yes | - An analysis to determine missing at random not conducted. |
| Halcomb et al. (2020) | Yes | Yes | Yes | Can’t tell | Yes | - An analysis to determine missing at random not conducted. |

Supplementary Table 2. Critical Appraisal Results Using Mixed Methods Appraisal Tool (MMAT) Version 2018 for Qualitative Research

| Author  (Year) | Is the qualitative approach appropriate to answer the research question? | | Are the qualitative data collection methods adequate to address the research question? | Are the findings adequately derived from the data? | Is the interpretation of results sufficiently substantiated by data? | Is there coherence between qualitative data sources, collection, analysis and interpretation? | Limitations |
| --- | --- | --- | --- | --- | --- | --- | --- |
| Akbar et al. (2022) | Yes | | Yes | Yes | Yes | Yes |  |
| Halcomb et al. (2020) | Yes | | Yes | Can’t tell | Yes | Yes | - Limited description of thematic analysis methods including specific codes, categories and themes. - Limited information on the analysis methods of quantifying the qualitative data. |
| James et al. (2021) | Yes | | Yes | Can’t tell | Yes | Yes | - No information on qualitative rigor and trustworthiness. |
| Lee et al. (2021) | Yes | | Yes | Yes | Yes | Yes |  |
| Martins et al. (2022) | | Yes | Yes | Can’t tell | Can’t tell | Yes | - Limited description of data analysis. - The quotes were written in Spanish. |
| Mizumoto et al. (2022) | | Yes | No | Can’t tell | Yes | Yes | - Data collection was conducted in participants’ workplace and during duty hours. - Prone to biased interpretation because the author, an employee at the data collection site, collected and analyzed the data. - No information on qualitative rigor and trustworthiness |
| Nilsen et al. (2022) | | Yes | Yes | Yes | Yes | Yes |  |
| Russsels et al. (2022) | | Yes | Yes | Yes | Yes | Yes |  |
| Yodsuban et al. (2023) | | Yes | Yes | Yes | Yes | Yes |  |
